# Protocol for a cluster randomized controlled trial among young people and their peers to reduce substance use and inequitable gender beliefs in Southern Africa

**DOI:** 10.1186/s13722-026-00718-7

**Published:** 2026-10-14

**Authors:** Tara Carney, J. Anitha Menon, Beatrice Chiyokoma, Kudakwashe Madzeke, Giuliana Sanchez, Kim Johnson, Mathias Basera, Aline Berweger, Laura Ruckstuhl, Sina Hansen, Lisa Hofer, Jennifer M. Belus

**Affiliations:** 1 Mental Health, Alcohol, Substance Use and Tobacco Research Unit, South African Medical Research Council, Parow, South Africa; 2 Department of Psychiatry and Mental Health, University of Cape Town, Rondebosch, South Africa; 3 School of Psychology, Curtin University, Dubai, United Arab Emirates; 4 School of Public Health, University of Zambia, Lusaka, Zambia; 5 SolidarMed, Lusaka, Zambia; 6 SolidarMed, Masvingo, Zimbabwe; 7 Division of Clinical Epidemiology, Department of Clinical Research, University Hospital Basel, University of Basel, Basel, Switzerland; 8 SolidarMed, Lucerne, Switzerland; 9 Department of Clinical Research, University Hospital Basel, University of Basel, Basel, Switzerland; 10 Department of Psychology and Department of Medicine, University of Zurich, Zurich, Switzerland

**Keywords:** Substance use, Gender norms, Adolescents and young adults, Peer invervention, Southern Africa

## Abstract

**Background and aims:**

Substance use and the internalization of traditional gender norms, or inequitable gender beliefs, are co-occurring risk behaviors for poorer health and well-being in young people in Southern Africa. Addressing the social and structural factors that reinforce substance use and inequitable gender beliefs, including peer influence and socioeconomic adversity, such as lack of education or employment, is necessary to support sustainable change in these behaviors. However, most existing interventions focus only on changing individual behavior. This study will therefore aim to address this limitation by testing the efficacy of an intervention to reduce substance use and promote equitable gender beliefs, delivered to peer groups and embedded within existing vocational training (VT) programs.

**Design:**

Two-arm cluster randomized controlled superiority trial with assessments at baseline, 12-week, and 24-week follow-up.

**Setting:**

Selected VT programs in Zambia, Zimbabwe, and South Africa.

**Participants:**

Sixty-six peer groups (*N* ~ 264 participants) will be recruited, comprised of an index case plus 2 to 5 peers aged 16–24 years old, who use substances together weekly, or binge drink at least monthly, and enrolled in eligible VT programs.

**Intervention and comparator:**

Peer groups will be randomly allocated [1:1] to receive either: (i) the standard VT program plus Peer Education for Gender Inclusion and Substance Use in Southern Africa (PEGISUS), an 8-session interactive intervention delivered by VT program staff to peer groups or (ii) standard of care (SOC), namely the standard VT program and a referral for substance use services.

**Measurements:**

The primary outcome is the between-group (PEGISUS vs. SOC) mean difference in change in the total substance use involvement score on the ASSIST (Alcohol, Smoking, and Substance Involvement Screening Test) from baseline to the 12-week follow-up. Secondary outcomes compare the between-group mean difference in (1) other substance use scales on the ASSIST and at other timepoints (i.e. 24-week follow-up) (2); change in gender equitable beliefs using the GEMS (Gender Equitable Men’s Scale) from baseline to the 12-week and 24-week follow-up; and (3) VT program attendance and completion rates, employment status, and income earned at the 24-week follow-up.

**Comments:**

This will be the first known study in Southern Africa to assess the efficacy of an intervention that addresses substance use and gender equity beliefs among young people and their peers delivered in VT programs. PEGISUS will address socioeconomic adversity and offer a potential pathway to sustainable delivery by embedding the intervention within existing VT programs. If unhealthy risk behaviors are reduced, and employment opportunities are improved, this can lead to improved well-being in the life trajectories of these young people.

**Trial registration:**

Clinicaltrials.gov Trials identifier: NCT06489899. Approved on July 8, 2024. url: https://clinicaltrials.gov/study/NCT06489899?term=NCT06489899?rank=1. Pan African Clinical Trials identifier: PACTR202502629432188. Approved February 14, 2025. url: https://pactr.samrc.ac.za/TrialDisplay.aspx?TrialID=33370.

**Supplementary Information:**

The online version contains supplementary material available at https://doi.org/10.1186/s13722-026-00718-7.

## Introduction

The African continent has the highest proportion of young people globally, with over half of its population under 24 years of age [1]. Unfortunately, young people face substantial challenges, including unemployment, fragile health systems, political instability, and climate-related stressors, all of which pose long-term threats to their health and well-being [2–5]. To deal with these challenging realities, substance use is often used as a coping strategy [6–8], with an estimated 20 to 40% of young people reporting use of at least one psychoactive substance [7, 9]. Early substance use increases the risk of mortality and morbidity across the lifespan [10, 11] and is associated with a number of adverse outcomes including HIV risk [12, 13], traffic accidents [14], intimate partner violence [15], unemployment [16, 17], and transition to substance use disorders [18]. Early intervention is therefore essential to improve life-long health and well-being.

In many Southern African contexts characterized by poverty, substance use may provide short-term reward for young people in settings characterized by both limited opportunities for alternative sources of positive reinforcement and lack of hopefulness about the future [19, 20]. Although evidence-based, early interventions for substance use in young people exist [21–23], access remains limited, with an estimated 95% of those in need not receiving care [24]. In addition, most existing interventions focus on the individual, neglecting broader social and environmental factors that contribute to substance use [25], including the role of peers in substance use initiation and escalation [25, 26]. This highlights the urgent need for innovative approaches to substance use and its complex drivers.

Traditional gender norms are similarly established early in life and reinforced throughout adolescence, shaping expectations and contributing to long-lasting unequal outcomes for men and women, including in Southern African countries [27, 28]. The internalization of these traditional gender norms, or inequitable gender beliefs, frequently co-occurs with substance use [29, 30], and is similarly associated with engaging in intimate partner violence [15, 30–32], risk of sexually transmitted infections and HIV [33], and mental health conditions [28]. Targeting these beliefs alongside substance use in an integrated manner may therefore target shared underlying drivers of overall health and well-being for young people.

Educational achievement and employment is a key protective factor against substance use [34] among other health-related risk behaviors for young people [34, 35]. However, structural barriers contribute to low rates of school completion and persistently high youth unemployment in the region. For example, 35% of youth aged 15 to 24 in South Africa are neither employed nor in school [36]. Vocational training (VT) programs provide an alternative pathway to improve employment and economic opportunities for young people by equipping them with practical, marketable skills [37, 38]. However, substance use and inequitable gender beliefs may jeopardize these benefits through their associations with school dropout, unemployment, and other adverse outcomes [17, 18, 39]. Addressing these issues in among VT participants may therefore help maximize the interlinked health and economic benefits that VT programs offer.

Together, substance use and gender equity beliefs, influenced by structural factors, can negatively influence young people’s health and well-being. Peers play an key role in reinforcing both the health promoting and risky behaviors of young people, including substance use and gender equity beliefs [13, 32, 40]. A recent review that looked at the role of peers in mental health interventions found that they have only been included in a limited number of studies, all conducted in high-income countries with even less focusing on substance use outcomes [41]. While a recent study including friendship groups among young adults in a South African setting showed a decrease in certain interpersonal violence and gender practices, alcohol was the sole substance measured and a significant decrease found in female friendship groups only [42], In addition, those directly targeting peer groups in VT programs have not been widely tested in low- to middle-income countries (LMICs) [21, 23, 26, 40].

## Objectives

### Primary

To test the efficacy of PEGISUS (Peer Education for Gender Inclusion and Substance Use in Southern Africa) on total substance use involvement score, in comparison to standard of care (SOC) at the 12-week follow-up timepoint. PEGISUS is an 8-session intervention targeting substance use and gender equity beliefs, delivered to peer groups aged 16 to 24 and embedded into existing VT programs.

### Secondary

To evaluate the efficacy of PEGISUS in comparison to SOC in terms of:

1. Change in other substance use scales and later timepoints (i.e., 24-week follow-up).
2. Change in self-reported gender equity beliefs from baseline to the 12- and 24-week follow-up.
3. VT program attendance and completion rates, employment status, and income earned at the 24-week follow-up.

We hypothesize that PEGISUS is superior to SOC on the primary and secondary endpoints.

## Methods

### Design and setting

The study is a two-arm cluster randomized superiority trial, implemented in three countries, and corresponds to a Stage III trial testing a behavioral intervention [43]. It will take place within partnering VT programs in Zambia, Zimbabwe, and South Africa that serve young people in economically disadvantaged communities, where unemployment is common and substance use, traditional gender norms, and gender-based violence are prevalent.

In Zambia, this will include multiple communities located in Chibombo and targeted vulnerable communities in the capital city Lusaka. Zambia has one of the highest rates of frequent alcohol use among young people compared to other low-to middle-income country settings [44] and 29% of young people aged 15 to 24 are either unemployed or not in school [45].

In Zimbabwe, the study will take place in the urban communities of Masvingo, which is in the 30th percentile globally for indicators of overall human development, including education, income, and health [46, 47]. Substance use is a growing public health concern among young people and is linked to poverty, school dropout, violence and suicide, especially in urban areas [48]. Thirty-six percent of young people aged 15 to 24 are neither employed nor in school [49].

In South Africa, the study will take place across multiple economically disadvantaged township communities, in the Cape Town area. There are high levels of poverty, crime, mental health issues, and social instability in the study communities [50], which are closely linked to high levels of substance use [51, 52]. Over a third of young people aged 15–24 are neither employed nor in education [53].

### Participants

#### Inclusion criteria for VT programs

VT programs provide essential skills training for employability and entrepreneurship for young people, who may not have completed formal education. These programs must meet the following inclusion criteria to be selected as study partners (1): enroll individuals of all genders aged 16–24 years old from economically disadvantaged communities (2); do not require participant high school completion (3); willing to enroll young people with current substance use (4); offer at least two different training program options not restricted by participant gender identity, allowing participants to choose a content domain of interest (5); include at least some business skills (e.g., numeracy) (6); provide a certificate of completion at the end of the program (7); willing to embed a brief substance use and gender equity curriculum into the institution’s existing program; and (8) located in a setting accessible to young people in the communities where recruitment takes place.

#### Inclusion criteria for young people

Study participants will be recruited through index cases who subsequently recruit 2 to 5 of their peers. Inclusion criteria for all participants (i.e., both index and peers) are as follows (1): aged 16–24 years old (2); self-reported weekly substance use or at least monthly report of binge drinking with peers over the previous three months (3); comfortable communicating in the predominant local language(s) or in English (4); live in the target communities served by the VT program and plan to live there until the VT program is complete (up to 12 months) (5); meet the specific entrance criteria of the VT program and willing to commit to participation in the VT program for its entire duration (6); interested in participating in the PEGISUS intervention and willing to have sessions audio/video-recorded (if peer group is allocated to this arm); and (7) identify 2 to 5 of their peers with whom they use substances to join the study (only relevant for the index participant).

#### Exclusion criteria for young people

Participants will be excluded if they meet any of the following criteria (1): untreated major mental illness that interferes with study participation (2); currently receiving psychological treatment for substance use (3); enrolled in a VT or other skills development program that site principal investigators (PIs) deem non-compatible with this study; or (4) unable to provide informed consent/assent.

### Treatment groups

#### Standard of care (SOC)

Peer groups randomized to SOC will receive the standard VT program offered through the partner organization, which may include existing training that supports uptake of healthy lifestyles (e.g., nutrition, exercise, relationships), and a referral for substance use services. This was selected as the control arm to compare the PEGISUS intervention with existing practices in the real-world context of VT programs. The VT program partner in each country is described below.

In Zambia, Build It International is an international development charity that has existed in the country since 2007 and continues to develop effective ways to help reduce unemployment and poverty through VT construction programs, such as bricklaying, carpentry, painting, plastering, and tiling. They enroll up to 250 students per year [54]. In Zimbabwe, Masvingo Polytechnic is a state-owned technical college that offers a variety of potential courses to choose from, including electrical engineering, welding, guidance counselling, motor mechanics, and hotel and catering. Masvingo Polytechnic has the capacity to enroll up to 200 students per year [55]. In South Africa, the VT program is offered through the Sozo Foundation, a nonprofit organization that provides training in six different vocational areas, including nutrition, hairstyling, and coding. They have the capacity to enroll up to 120 students per year [56].

#### Intervention

Peer groups randomized to the PEGISUS intervention will participate in the intervention sessions together. PEGISUS is an 8-session manualized intervention and builds on two evidence-based interventions: RAD-PAL for substance use [57] and Manhood 2.0 for gender equity beliefs [58]. To develop the intervention, we conducted formative work constituting 12 focus group discussions (*n* = 97 participants) with young people from the target population, obtained feedback from a local youth advisory board in each country, and conducted a pilot study with 18 peer groups (*n* = 57 participants) to evaluate the preliminary acceptability and feasibility of the intervention content and implementation.

Trained interventionists who are staff members within their VT program will deliver PEGISUS intervention sessions, which are estimated to last approximately 1.5 hours and be activity-based. All members of the peer group receive the sessions together and participant enrollment type (i.e., index vs peer participants) is not relevant for any component of intervention delivery. The peer-based group sessions cover topics related to substance use, gender, and building healthy relationships and support, with all material and activities geared towards the peer group working together. (see Table 1 for an overview of the topics covered in each session). Participants will also receive a handbook containing important information covered in the sessions, including reflection activities that are completed individually. Intervention sessions will be delivered according to the VT program’s own schedule, but peer groups cannot receive more than two sessions per week. Intervention sessions must be delivered before the 12-week assessment, which will occur ≥ 8 to 20 weeks post randomization.

**Table 1:** Outline of PEGISUS sessions

Session	Activity name and description
1	1. 1 Introduction to PEGISUS and Icebreaker 1. 2 Values Clarification and Group Name 1. 3 Peer Group Strengths
2	2.1 Psychoeducation for Substance Use Activity 2.2 Decisional Balance Activity 2.3 Envision the Future
3	3.1 What is Gender? 3.2 What are Gender Norms?
4	4.1 Understanding Gender Roles 4.2 Stepping Outside of Gender Boxes
5	5.1 Expressing My Emotions 5.2 Substance Use Triggers 5.3 Healthier Alternatives to Substance Use
6	6.1 Problem-Solving Tool 6.2 Problem-Solving to Fight Back Against Gendered Expectations and Substance Use Triggers
7	7.1 Circles of Influence 7.2 Social Network
8	8.1 Brainstorming Goals 8.2 Readiness to Change and Workshop Reflection

### Sample size

The assumed standardized effect size is based on a meta-analysis of brief behavioral interventions for substance use in young people [59], which showed motivational interviewing-based interventions were more effective than SOC to reduce substance use, with a standardized net mean difference of −0.5 units (95% CI −1.0 to 0.0). In this study, our primary endpoint measure for substance use will be the change in the total substance use involvement score on the screening tools selected (Alcohol, Smoking, and Substance Involvement Screening Test (ASSIST))from baseline to 12 weeks. The primary outcome is the between-group mean difference of the change in the total substance use involvement score from baseline to 12 weeks (scores ranging from 0 to 33 for adolescents aged 16–17 years on the ASSIST-Y and; 0–39 per substance for young adults aged 18–24 years on the ASSIST). Our pilot data suggest total substance use involvement scores can be in the range of up to 100 and 150 respectively. We assume a standard deviation of 17 points based on pilot data testing the PEGISUS intervention. Assuming equal standard deviations in both groups (no data available for SOC), this translates into a net mean difference of −8.5 points on the ASSIST total substance use involvement score at the 12-week assessment between the two groups, in favor of the PEGISUS intervention group.

Clusters will be recruited with a cluster size of 3 to 6 participants (index case and 2 to 5 peers). We assume no dropout of complete clusters but approximately 20% dropout of individual participants, and a average cluster size of 4 participants. The intra-class correlation within peer-groups for the total substance use involvement score from our pilot data was 0.02, but we will increase it for the trial to 0.08 for a more conservative estimate. With these assumptions (standardized net mean difference = −0.5, ICC = 0.08, average cluster size of 4), a two-sided significance level of 0.05 and a power of 95%, a total of 66 clusters (33 clusters in each arm) will be needed to detect a difference in the total substance use involvement change score on the ASSIST between PEGISUS and SOC. With an average cluster size of 4, this translates to approximately 264 participants in total. We will aim for an equal distribution of the 66 clusters among the three countries.

### Procedure

#### Recruitment and screening

In each setting, young people will be recruited from communities viewed as economically disadvantaged that are geographically accessible or specifically targeted by the VT programs because of known high levels of risk behaviors, including substance use. Recruitment will take place in a variety of settings, including informal substance use venues, through community organizations that work closely with young people, and general community venues where young people congregate, such as libraries (where free Wi-Fi is accessible). We will also directly recruit participants who have applied to the VT program and meet eligibility criteria.

Trained study staff will complete in-person screening with an identified index participant, who then has up to one week to recruit peers who are screened either in-person or telephonically, depending on their location. Once all identified peers of the index participant have been screened, (index participant plus 2 to 5 peers), those in the peer group who meet eligibility criteria will be invited to consent.

#### Informed consent

Eligible peer groups will be required to consent to study participation in-person prior to any study activities. Participants 18 years of age or older can provide self-consent. Minor participants aged 16 and 17 years old must be accompanied by a main caregiver (parent or guardian), who will need to provide informed consent before assent is obtained from potential minor participants.

Trained staff will conduct the informed consent process, either in English or the local language (depending on participant preference). Research staff will explain the nature of the study, its purpose, the procedures involved, the expected duration (including the length of the VT program), potential risks and benefits, and any discomfort the study may entail. Each potential participant will then be informed that study participation is voluntary and that they may withdraw from the study at any time, which will not affect their subsequent services and treatment, including participation in the VT program. Finally, potential participants will be made aware that PEGISUS intervention sessions will be audio-recorded to ascertain correct delivery of the intervention. During the consenting process, participants will be given the opportunity to individually ask questions and address any concerns they may have before signing the consent forms. Following this, they will be provided with a signed copy containing contact details for any additional questions or concerns that may arise during their involvement in the study.

#### Randomization

**Fig. 1:**
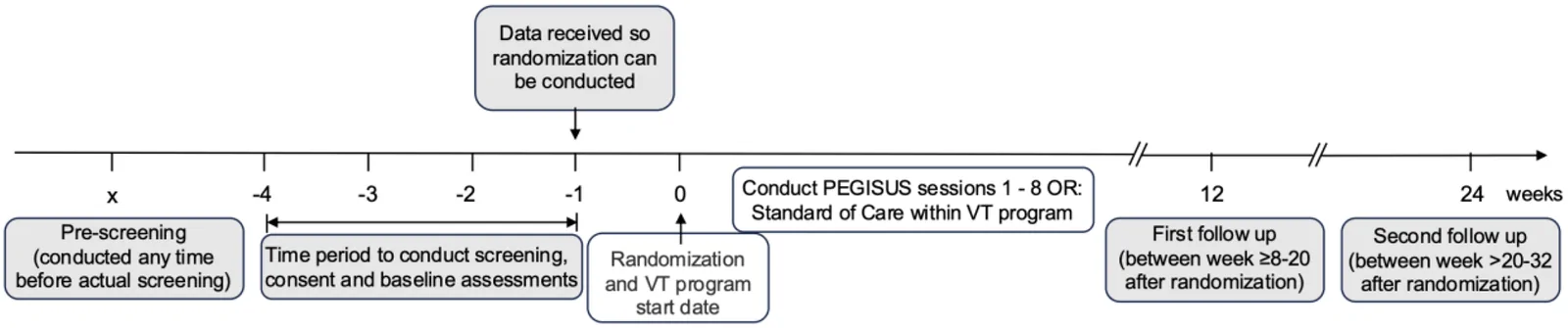
Timeline of study events. *VT = vocational training

After consenting and completing the baseline assessment, peer groups will be randomized 1:1 to either receive (1) the standard VT program plus the PEGISUS intervention or (2) SOC. The randomization allocation will be conducted using REDCap, pre-programmed with the correct concealed allocation by an independent data manager who has no participant contact following the completion of baseline assessments by eligible peer groups. Randomization will also be stratified by country to ensure balance of treatment arms within and across countries. All study staff members who work directly with participants, including study investigators, will be blinded to the randomization sequence. The data manager then will enter the peer group ID number into REDCap and click “randomize” to reveal the group’s set allocation. The randomization results for each peer group are communicated to the site coordinators in each country, who in turn communicate the results to the VT program interventionists as well as directly to participants within each peer group (see Fig. 1 for randomization timeline). Blinding of participants and interventionists will not be possible due to the intervention and the study design, as is the case with most behavioral interventions.

### Measures

#### Primary outcome

The primary endpoint is the change in self-reported substance use from baseline to the 12-week follow-up using the total substance use involvement score on the Alcohol, Smoking and Substance Involvement Screening Test (ASSIST), a validated, 8-item tool with recognizable cutoffs for low, moderate-, and high-risk use over the past 3 months across ten substances [60]. Participants aged 16–17 years will complete the ASSIST-Y, an adapted version with age- and substance-specific risk cutoff scores [61]. The total substance use involvement score is calculated by summing scores across all substances except tobacco, for a total of nine substances, with higher scores indicating greater substance use severity and related problems. The total substance use involvement score is calculated by summing scores across all substances except tobacco, for a total of nine substances. Each substance has a possible range from 0 to 39 on the ASSIST and a range of 0–33 on the ASSIST-Y. Our pilot data suggests total substance use involvement scores in the range of 8–142.

#### Secondary outcomes

There will be two additional secondary endpoints related to substance use (1): change in the total substance use involvement score from baseline to the 24-week follow-up and (2) change in specific substance use scores on the ASSIST from baseline to the 12-week and 24-week follow-up.

We will also evaluate changes in self-reported gender equity beliefs from baseline to 12- and 24-week follow-up timepoints using the 23-item Gender Equitable Men’s Scale (GEMS) [62, 63], with higher scores indicating more equitable beliefs.

Finally, at the 24-week follow-up, we will assess four VT-related secondary endpoints (1): duration of VT attendance (months) (2), program completion (yes/no) (3), employment status, and (4) income earned. Data will be obtained from VT records, where available, and supplemented by self-report.

### Participant timeline

**Fig. 2:**
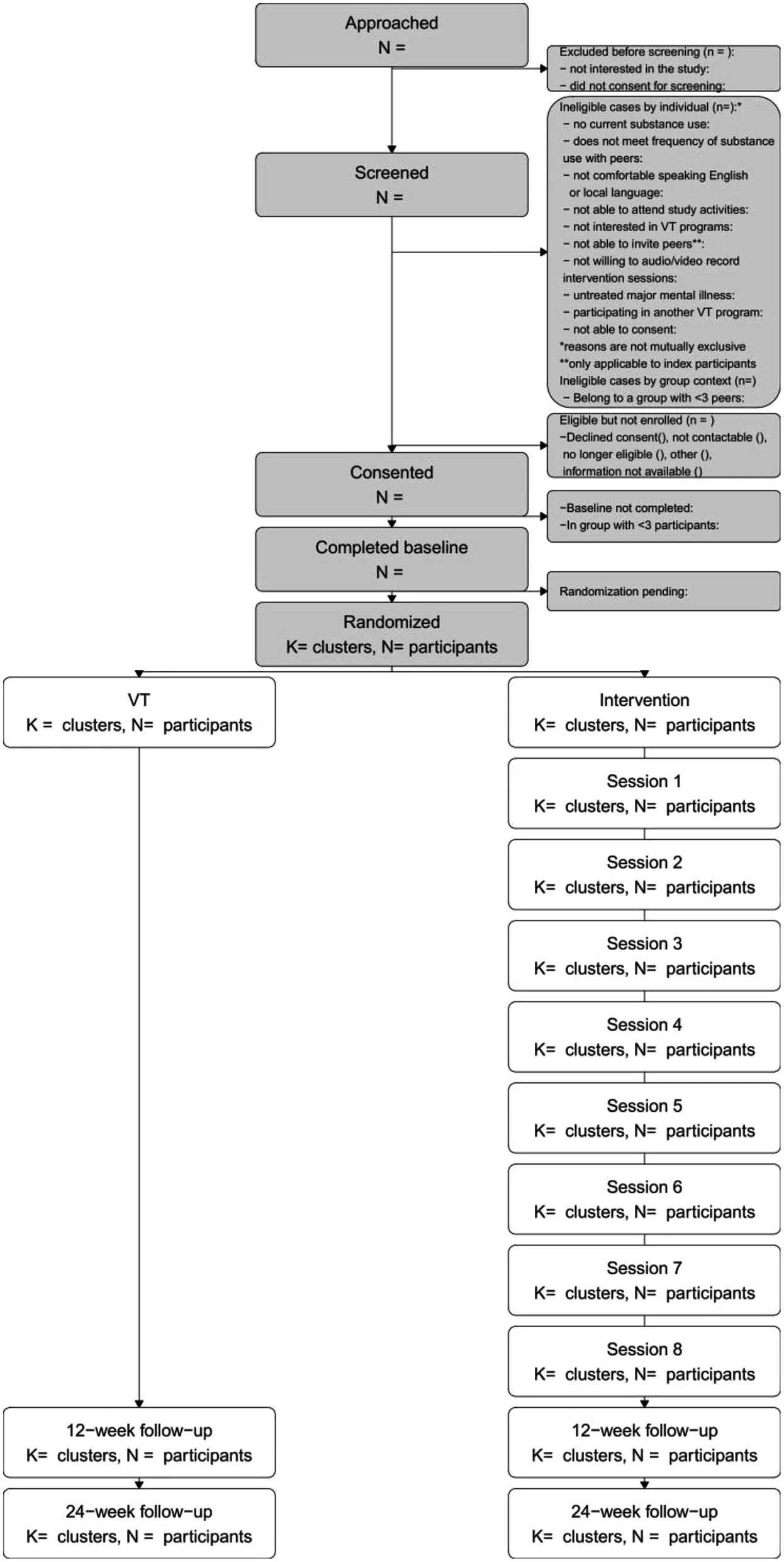
Flow chart of the study

Screening, consenting, baseline assessments, and study intervention allocation will take place in relation to each VT program’s enrollment dates. Screening, consenting, and baseline assessments of participants must occur over a 3-week period, which begins approximately 4 weeks before the VT program commences. Study staff can will conduct pre-screening prior to this window to identify potentially eligible peer groups; but must re-screen these peer groups within the window to ensure current eligibility. One week before the VT program begins, baseline assessment data completion will be checked, peer groups randomized following the completion of baseline assessments by eligible peer groups, and treatment arm allocation communicated. Follow-up assessments will be conducted at approximately 12-weeks (range ≥ 8 weeks to 20 weeks) and 24-weeks (range > 20 weeks to 32 weeks) post-randomization. Participants will be reimbursed for their time with a grocery voucher for each research appointment receiving an incentive to the value approved by each country’s ethical guidelines, ranging in value from 10 to 13 USD at baseline, 10–16 USD at 12-weeks, and 10–18 USD at 24-weeks. Detailed contact information will be collected from participants during their baseline appointment and research staff will actively track participants to minimize attrition at 12- and 24-week follow-up. See Table 2 for the SPIRIT schedule of study activities and outcome measures and Fig. 2 a visual representation of the study procedures.

**Table 2:** Schedule of enrolment, interventions, and assessments

	Screening	Baseline	Randomization	Treatment	Follow-up Assessments	
TIMEPOINT	-4 weeks to −1 week	-4 weeks to −1 week	0	0 -12 weeks	12 weeks	24 weeks
ENROLMENT:						
Eligibility screen	X					
Written informed consent		X				
Allocation			X			
INTERVENTIONS:						
PEGISUS						
SOC						
ASSESSMENTS:						
Substance use		X			X	X
Gender equitable beliefs		X			X	X
Vocational skills and employment		X				X

### Intervention delivery

Interventionists will constitute employees at the VT program partner and hold various positions within the organization, including wellness and skill trainers and student coordinators. Each site has between 2 to 5 trained interventionists, selected on the following criteria: previous experience with facilitating groups, working with young people, employed at the VT program for at least six months, and planned future employment for the next year.

We will ensure all interventionists are trained in good clinical practice and receive in-person and online training that covers PEGISUS-specific topics, including substance use psychoeducation, motivational interviewing, understanding the difference between gender and sex, as well as specific content for each intervention session. An initial 5-day training was conducted to prepare for the pilot test phase of the intervention. Feedback from the pilot study (audio recorded fidelity checks and interventionist feedback) informed a subsequent 2-day online training on topics related to general facilitation skills, gender, handling distressed participants, and bringing the intervention to a close. This training included both didactic information and role play practice. New interventionists joining the team during the study will be required to watch recordings of the initial and follow-up training sessions.

All PEGISUS intervention sessions will be audio-recorded, and interventionists receive two levels of supervision and technical support. First, interventionists in each country will receive regular technical group support delivered by a trained member of the research team. This will last approximately one hour and cover fidelity feedback on audio-recorded sessions, implementation issues (such as group engagement, time management, specific content questions), and referrals for participants who need additional clinical services. A fidelity checklist has been developed for each session based on the intervention manual. Each component or step of the intervention will be rated on a 3-point scale: 0 (did not complete the outlined criteria), 1 (satisfied some outlined criteria), or 2 (satisfied all outlined criteria). Moreover, each site has designated referral pathways to local clinicians who have agreed to consult on study cases. Audio recordings of intervention sessions will be uploaded to a secure, password-protected shared folder in each country before being deleted from audio recorder devices. These folders will only be accessible to specific staff members. They will be saved as specified by each country’s Good Clinical Practices guidelines (South Africa 10 years; Zambia 6 years; Zimbabwe 5 years after completion of data collection).

Secondly, interventionists will receive regular supervision from a clinically-trained individual, following guidelines for supervising non-specialist providers [64]. This includes review of intervention skills, challenges in implementation, and provision of emotional support for providers to prevent burnout.

### Data collection and management

Study assessments at baseline and follow-up timepoints follow similar procedures. Trained research assistants from the local community will first establish rapport with study participants to foster trust and then conduct interview-led data collection to obtain the most accurate data possible. Standardized questionnaires are programmed into REDCap, an electronic data collection and management software, which allows for direct data entry and real-time error checking [65]. Study research assistants who conduct baseline and follow-up assessments will be blinded to the intervention arm, as well as investigators. It will not be possible to blind participants to the behavioral intervention.

### Data analysis

Primary and secondary analyses will follow the Consolidated Standards of Reporting Trials (CONSORT) guidelines [66] and modified intention-to-treat principles (available participant data analyzed according to their randomization condition, regardless of whether assigned treatment is completed). Clusters of any size (after potential dropouts) will be included. Baseline characteristics will be described by study arm using summary statistics; no formal testing between arms will be performed.

The primary outcome (between-group mean difference of the change in the total substance use involvement score on the ASSIST from baseline to the 12-week follow-up), will be analyzed combining data from all three countries. It will be estimated using a linear mixed-effects model with cluster-specific random effects, adjusting for the baseline total substance use involvement ASSIST score, and covariates sex and age. If model assumptions are violated, we will choose a more appropriate error distribution. The superiority of PEGISUS will be demonstrated if the lower bound of the 95% confidence interval for the mean difference between the groups is larger than 0. The primary and secondary analyses will be performed using complete-case analysis, as follow-up score(s) are necessary to calculate each participant’s change score. However, we plan to conduct a sensitivity analysis for the primary analysis in case more than 15 endpoints are missing (around 5% of the planned number of participants), where we will use multiple imputation methods to impute missing primary endpoints at 12-week follow-up.

As a secondary exploratory analysis we will subsequently evaluate whether there are differences in the intervention effect between the three countries by adding an interaction to the model between intervention and country. The interaction test will be carefully interpreted, as these tests are usually underpowered [67]. If the interaction is significant, a subgroup analysis for each country will be performed the same way as the primary analysis to provide the estimated intervention effect for each country.

The secondary endpoints will follow a similar analytic plan and will compare the between-group mean difference of the change in (1): substance use from baseline to the 24-week follow-up using the total substance use involvement score on the ASSIST (2), substance-specific versions of the ASSIST (e.g., score on alcohol use, cannabis use, etc.) from baseline to the 12-week and 24 week follow-up, and (3) gender equitable beliefs using the GEMS from baseline to both follow-up timepoints. At the 24-week assessment, we will also compare between-group mean differences in the VT program attendance and completion rates, employment status, and income earned.

A detailed statistical analysis plan will be finalized, dated, and signed prior to the completion of study follow-up assessments. Missing baseline and outcome data will be summarized by study arm. Our team’s previous clinical trials with similar populations show low rates of attrition (<10%), based on rapport built with participants and communication with participants [68, 69]. For the current study, we do not expect full clusters to drop out, as individual participants are able to continue participating in all study activities, even if all other peer group members drop out. Individual participants who drop out, miss a study assessment, or are lost to follow-up will not be replaced by new participants. This has been accounted for in the sample size estimate by the assumed average cluster size.

### Data safety and monitoring plan

Each study participant will be assigned a unique participant identification number per country, which is the only link between study-related data and personally identifying information (e.g., participant names, telephone numbers) collected, which will be stored locally in each country and only accessible to authorized study team members. Data collected through REDCap will be hosted by the Department of Clinical Research at the University Hospital Basel, in Switzerland. All researchers on the project will have access to the database via personalized login information, with data rights capabilities set according to the role of each research team member. The Swiss team will be responsible for data management (e.g., data checking, quality assessment) and will liaise with each country team weekly to ensure all data queries or data quality issues are addressed in a timely manner. All staff members undergo comprehensive training on the study’s standard operating procedures.

#### Adverse events

All serious adverse events (SAEs) should be documented and reported immediately (within a maximum of 24 hours) to each Site PI and Sponsor-Investigator of the study. The Site PI will evaluate the severity of the SAE according to the Common Terminology Criteria for Adverse Events grading system [70] and make a causality assessment of the event in relation to the study intervention according to the standards set by the International Conference on Harmonisation, topic E2A [71]. The Sponsor-Investigator will then review the severity and causality statements assigned by each Site PI.

If an SAE is “possibly,” “probably,” or “definitely” related to the study, the Site PI must report the event(s) to the local Ethics Committee within 48 hours. Participants who complete study activities with reported ongoing SAEs that are “possibly,” “probably,” or “definitely” related to the study intervention will be followed up until resolution or stabilization of the SAE, for up to 6 months post-study termination.

### Ethical considerations

This study has been approved by the following health research ethics committees: Zambian National Health Research Authority and University of Zambia Biomedical Research Ethics Committee (4988–2024), Medical Research Council of Zimbabwe (A/3076), and South African Medical Research Council (EC042-11/2023). It was also reviewed by the Ethikkommission Nordwest- und Zentralschweiz in Switzerland, who confirmed that the study met ethical and scientific standards for research with human subjects, but operates outside of the jurisdiction of the ethics board (AO_2023-00095). Substantial changes that are made to the study setup and study organization, the protocol, or other relevant study documents will be submitted to local ethics committees in each country for approval before implementation.

There are several important areas of ethical consideration for the study, given the focal topics of substance use and gender equity among young people in Zambia, Zimbabwe, and South Africa. During study participation, participants will be offered necessary referrals for mental health, substance use, or other health-related services. Since the study population includes members of a vulnerable population (i.e., minors under the age of 18, possible use of illegal substances), it is critical to protect these participants by ensuring that the appropriate parental consent is obtained before the assent process. Due to the sensitive nature of this study, we are aware that disclosing minor’s substance use could increase harm, and result in conflict or violence in the family. Therefore, the parental consent form will include the importance of addressing substance use risk among young people in general and not explicitly state the selection of young people based on a substance use threshold. Secondly, if any participant under 18 reports being abused, this will need to be reported to appropriate authorities alongside other reportable harms. All staff will be trained on procedures in how to assess the situation and take necessary steps to ensure participant safety.

Moreover, since the PEGISUS intervention will be delivered to peer groups, additional ethical considerations need to be taken into account. It is important that study participation is voluntary and that peers do not coerce participants to take part. Screening for the study will take place separately for individuals within peer groups, so that study staff can ascertain if individuals ares genuinely interested in the study. Peer groups randomized to the PEGISUS intervention arm may receive the intervention with other peer groups. When bringing together individuals of different backgrounds in a group setting and asking them to take part in activities related to substance use and gender beliefs, disagreements or conflicts may arise. To minimize this risk, group ground rules are set, and interventionists receive group facilitation training and ongoing supervision.

Ethical considerations were carefully guided by the legal and sociocultural sensitivities surrounding sexual and gender minority identities in Zambia and Zimbabwe. The penal code in these countries still criminalizes sexual acts between those of the same sex and does not recognize transgender and gender-fluid gender identities. As such, participants will be asked not to disclose these identities or any related information. Interventionists will also receive training to navigate such topics with cultural sensitivity, prioritizing participant safety and dignity while adhering to local legal frameworks.

### Dissemination plan

The study findings will be submitted to peer-reviewed journals and conference presentations and shared at relevant stakeholder meetings within each study country and internationally. All investigators and collaborators listed on the research protocol will have the opportunity to contribute to the publication of results. To facilitate capacity building and foster academic careers in Zambia, Zimbabwe, and South Africa, the study team commits to encourage and support publications by collaborators from these countries by providing mentorship and instrumental support (e.g., protected writing time).

In addition to academic outputs, our team is committed to disseminating the findings to local stakeholders in each country to share evidence and foster dialogue. This includes non-governmental organizations, Ministries of Health, and community leaders. We will also disseminate the findings directly back to the young people who were part of the study; strategies to do this are guided by the youth advisory boards in each country.

## Discussion

Young people in Southern Africa face numerous challenges, including high rates of unemployment, health systems under pressure, political instability, and increasing evidence of the global climate crisis that affects their health and well-being [2, 4–6]. Substance use is a common coping strategy for many young people [7–9], but few interventions address the broader social environment that reinforces substance use and other risk behaviors [22]. This paper describes the study protocol for an intervention to reduce substance use and increase gender equitable beliefs, delivered to young people and their peer groups within VT programs in Zambia, Zimbabwe, and South Africa. Findings from the study will add to the evidence base on novel intervention approaches to reduce substance use and address co-occurring risk behaviors for young people.

One of the ongoing challenges in the global field of substance use is the large treatment gap, particularly in LMICs, which is partially due to low awareness in recognizing unhealthy substance use [24]. From a public health perspective, alternative models of care are needed to make adequate inroads to reducing this chasm. The PEGISUS intervention, embedded within existing VT programs and delivered by VT program staff, offers an opportunity for young people to identify their own unhealthy substance use and a path to scaling the intervention, should it be effective. Our approach fits with a recent scoping review of community-based interventions for adolescent and adult mental health and substance use problems in Africa, which concluded that interventions delivered outside of healthcare facilities were generally acceptable, feasible, and possibly effective [72]. Substance use interventions for young people in Africa are most often school-based, but extant evidence suggests such interventions do not always lead to behavior change among those who already use substances, possibly because they universally target all learners [22]. In contrast, the PEGISUS intervention targets young people who already demonstrate at-risk substance use behaviors and includes young people who are not enrolled in traditional education programs or formally employed, which are a vulnerable population.

This trial has strengths and limitations that should be considered. Study strengths include the use of a cluster randomized design with real-life peer groups and embedding the intervention into VT programs, offering an avenue for future scale up and possible sustainability. In addition, implementing the study across three Southern African countries enables the collection of evidence on the intervention’s effectiveness in diverse yet comparable contexts, facilitating a more rapid identification of the conditions under which the intervention is most effective. Finally, the intervention simultaneously addresses two mutually reinforcing risk behaviors, substance use and inequitable gender beliefs, which may negatively influence the health and well-being of young people. However, study limitations include potential selection bias as index participants may by more likely to select peers who are similar to them in terms of risk behavior and therefore there may be a high level of homophily in the sample, and generalizability of type of substance use, social environment substances where substances are used and other risk-taking behavior. A further potential limitation is that such behavioral interventions make it impossible to blind participants to the intervention, which can lead to performance bias and influence study outcomes, especially if they are self-reported as they will be in this study. Finally, the fact that the secondary analysis of intervention effectiveness within country is exploratory and likely to be underpowered and the final follow-up assessment window is only 6 months is a limitation, meaning that it is not possible to evaluate longer-term impacts. This is particularly relevant for employment status, as job acquisition and continued employment may fall outside this time window for most participants.

## Conclusions

Overall, this study uses a rigorous, cluster randomized trial design to evaluate the PEGISUS intervention to address substance use and inequitable gender beliefs among young people aged 16 to 24 and their peers within existing VT programs. The intervention, developed with feedback from young people themselves, seeks to address at-risk young people living in disadvantaged communities in Zambia, Zimbabwe, and South Africa. Innovation in the study includes targeting real-world peer groups and embedding the intervention into existing VT programs. Taken together, this study seeks to address key behaviors during a period of vulnerability by helping young people develop healthy coping skills beyond substance use, adoption of equitable gender beliefs, and improved employment opportunities, which may lead to improved well-being throughout this developmental phase and in the life trajectories of these young people.

## Electronic supplementary material

Below is the link to the electronic supplementary material.

Supplementary Material 1

## Data Availability

A de-identified dataset used for the analysis of the outcomes described within this paper will be made available alongside the published main outcomes paper.

## Declarations

### Protocol version 1.0

4 March 2024 (full list of approvals and amendments in Supplementary Materials)

### Project status

The study is currently enrolling participants. The first participant was enrolled on 10th March 2025.
