# Supplementary material for: Protocol for a cluster randomized controlled trial among young people and their peers to reduce substance use and inequitable gender beliefs in Southern Africa
Source: Addict Sci Clin Pract. 2026 Oct 14;21:74. doi: 10.1186/s13722-026-00718-7 (PMC13663153; doi:10.1186/s13722-026-00718-7)
Supplement: Supplementary file 1 [file 13722_2026_718_MOESM1_ESM.docx]

**Supplementary Table** Full List of Ethics Approval and Amendment Dates

| Version | Date (DD/MM/YY) | Section(s) changed | Description of changes and rationale |
| --- | --- | --- | --- |
| 1.0 | 04/03/24 | -- | Initial version |
| 2.0 | 19/09/25 | Study synopsis | Clinicaltrials.gov identifier added.  Updates to the primary endpoint, statistical considerations, and number of participants sections, corresponding to the changes made within the protocol (see below). |
| 2.0 | 19/09/25 | 3.1.2 Primary and secondary endpoints | More specificity added to the primary and secondary endpoints. Protocol now indicates primary endpoint is the change in the total substance use involvement score on the ASSIST from baseline to 12-weeks.  Specified that one of the secondary endpoints is the change in specific substance use scores (e.g., alcohol use, cannabis use) on the ASSIST from baseline to 12- and 24-weeks follow-up. |
| 2.0 | 19/09/25 | 6.1.2 Determination of sample size | Updated assumptions for the sample size calculation to align with the specification that the primary endpoint is the total substance use involvement score on the ASSIST. Total planned sample size remains the same. |
| 2.0 | 19/09/25 | 6.1.3 Planned analysis | Clarified that the primary outcome is the between group mean difference of the change in the total substance use involvement score on the ASSIST for the entire sample.  Clarified planned sensitivity analyses to examine between country differences in the primary outcome. |
| 2.0 | 19/09/25 | 6.1.5 Handling of missing data and drop-outs | Clarified expectations that no full clusters are expected to drop out.  Clarified the conditions under which planned sensitivity analyses to impute missing data on the primary endpoint would be conducted. |
